# Platelet P2Y12 Is Involved in Murine Pulmonary Metastasis

**DOI:** 10.1371/journal.pone.0080780

**Published:** 2013-11-13

**Authors:** Yanhua Wang, Yueping Sun, Ding Li, Lin Zhang, Kemin Wang, Yong Zuo, T. Kent Gartner, Junling Liu

**Affiliations:** 1 Department of Biochemistry and Molecular Cell Biology, Shanghai Key Laboratory of Tumor Microenvironment and Inflammation, Shanghai Jiao Tong University School of Medicine, Shanghai, China; 2 Department of Biology, University of Memphis, Memphis, Tennessee, United States of America; Cincinnati Children's Hospital Medical Center, United States of America

## Abstract

The involvement of platelets in tumor progression is well recognized. The depletion of circulating platelets or pharmacologic inhibitors of platelet activation decreases the metastatic potential of circulating tumor cells in metastasis mouse models. The platelet ADP receptor P2Y12 amplifies the initial hemostatic responses activated by a variety of platelet agonists and stabilizes platelet aggregation, playing a crucial role in granule secretion, integrin activation and thrombus formation. However, the relationship between P2Y12 and tumor progression is not clear. In our study, the Lewis Lung Carcinoma (LLC) spontaneous metastatic mouse model was used to evaluate the role of P2Y12 in metastasis. The results demonstrated that P2Y12 deficiency significantly reduced pulmonary metastasis. Further studies indicated that P2Y12 deficiency diminished the ability of LLC cells to induce platelet shape change and release of active TGFβ1 by a non-contact dependent mechanism resulting in a diminished, platelet-induced EMT-like transformation of the LLC cells, and that transformation probably is a prerequisite of LLC cell metastasis. Immunohistochemical analyses indicated an obvious P2Y12 deficiency related attenuation of recruitment of VEGFR1+ bone marrow derived cell clusters, and extracellular matrix fibronectin deposition in lungs, which presumably are required for pre-metastatic niche formation. In contrast to the LLC cells, non-epithelial melanoma B16 cells induced platelet aggregation in a cell number and P2Y12-dependent manner. Also, a platelet induced EMT-like transformation of B16 cells is dependent on P2Y12. In agreement with the LLC cell model, platelet P2Y12 deficiency also results in significantly less lung metastasis in the B16 melanoma experimental metastasis model. These results demonstrate that P2Y12 is a safe drug target for anti-thrombotic therapy, and that P2Y12 may serve as a new target for inhibition of tumor metastasis.

## Introduction

The involvement of platelets in tumor progression and metastasis in mouse models has been recognized for decades [1,2]. The depletion of circulating platelets, or pharmacologic inhibitors of platelet activation decrease the metastatic potential of circulating tumor cells in mouse models of experimental metastasis [3-5]. Platelet receptors, such as GPIb/IX/V, P-selectin and integrin αIIbβ3, can promote the progression and metastases of various types of tumors, and are potential targets for further clinical study [6-8]. Additionally, the control of the release of angiogenic proteins from platelets represents an approach to the control of blood vessel proliferation within the tumor microenvironment [9]. A recent study reported that high levels of TGF-β1 were released from platelet α-granules, and that the TGF-β1 could induce an epithelial-mesenchymal cancer cell transition (EMT) [10,11], a transient and reversible process that promotes cancer cell motility, invasiveness, and metastasis [12-14]. However, the role of platelets in tumor metastasis is not limited to those effects. Several studies suggested that thrombophilia caused by pro-coagulant mediators released in response to tumors may protect from external expansion and cancer dissemination [15], and deficiency of certain platelet surface receptors even resulted in enhanced experimental tumor metastasis [16].

The platelet ADP receptor P2Y12, which was first identified in 2001 [17], plays a prominent role in amplifying platelet activation, aggregation and thrombus formation. Previous studies using patients and mice with dysfunctional P2Y12 have demonstrated that P2Y12 plays a crucial role in platelet storage granule secretion, P-selectin expression [18,19], integrin GPIIb-IIIa activation [20], and thrombus formation [21,22]. Consequently, the P2Y12 inhibitor clopidogrel is widely used clinically to treat coronary artery, peripheral vascular and cerebrovascular diseases [23,24]. Recently, several publications analyzed the TRITON-TIMI 38 clinical trial (that tested the efficacy and safety of Prasugrel, a newly FDA approved thienopyridine P2Y12 inhibitor) revealing an increase in multiple types of solid tumors with Prasugrel use [25-27], casting doubt on the safety of anti-platelet therapy targeting P2Y12. Therefore, we investigated the role of P2Y12 in tumor metastasis.

Our investigation of the effect(s) of P2Y12 on tumor metastasis and growth was done using two pulmonary metastasis models. The results showed that the absence of P2Y12 significantly decreases pulmonary metastasis in mice. Further analyses revealed that P2Y12 deficiency in platelets decreases cytokine release resulted in significantly less EMT-like morphologic change of tumor cells, as well as diminished formation of the pre-metastatic microenvironment.

## Materials and Methods

### Ethics Statement

The animal research was approved by the Shanghai Jiao Tong University School of Medicine Animal Care and Use Committee (Approve No. SYXK2008-0050).

### Materials

ADP, Apyrase, PGE1 and Calcein were purchased from Sigma-Aldrich. Histostain-Plus Kits was purchased from Invitrogen. α-thrombin was from Enzyme Research Laboratories (South Bend, IN). Collagen was from Nycomed Arzneimittel (Munich, Germany). The mouse TGF-β1 ELISA Kit was from BioMart (Shanghai, China). The recombinant TGF-β1 was from Peprotech (NJ, USA). The anti-TGFβ1 blocking antibody was from R&D system (MN, USA). The rabbit monoclonal anti-VEGFR1 antibody was from Epitomics (Burlingame, CA), and the polyclonal rabbit anti-fibronectin antibodies were from Abcam (Boston, MA).

### Mice

P2Y12^−/−^ mice [22] and littermate P2Y12^+/+^ control mice (Wild Type, WT) on a C57BL/6J genetic background were 6-8 weeks old when used for experimentation, the groups were sex-, weight- and age-matched. The mice were anesthetized by intraperitoneal injection of pentobarbital before tumor cell inoculation or surgery.

### Cell culture and generation of cell lines stably expressing Ds-Red

B16 melanoma cells and Lewis lung carcinoma (LLC) cells were maintained as monolayer cultures in high glucose DMEM supplemented with 10% Fetal Bovine Serum, 1mM sodium pyruvate, 4mM L-glutamine and 100U/ml penicillin, 100μg/ml streptomycin in an atmosphere of 5% CO2 in cell holder at 37°C. Lentiviral vectors pLVX-DsRed coding for the Ds-Red protein (Clontech) were transfected into B16 and LLC cells as previously reported [28]. B16 melanoma cells and LLC cells were purchased from the Cell Bank of Type Culture Collection of Chinese Academy of Sciences, Shanghai, China.

### Pulmonary metastasis mouse models

Cells harvested from sub-confluent cultures with 0.05% trypsin-EDTA, were neutralized with medium containing 10% fetal bovine serum, then washed for 3 times in PBS and re-suspended in PBS. Single-cell suspensions of greater than 90% viability were used for the study of pulmonary metastasis in mice.

For the spontaneous pulmonary metastasis studies, 2x10^6^ LLC cells in 200μl PBS were injected intradermally into each of the P2Y12^−/−^ and WT mice. By day 14, the complete primary tumors were surgically removed from each mouse to improve animal survival. One month after surgery, the mice were sacrificed, the lungs removed, rinsed with PBS and observed under the dissecting microscope (Nikon). Then, the lungs were weighed, paraffin-embedded, sectioned and stained with hematoxylin and eosin. Stained pulmonary sections were observed under the light microscope (Zeiss).

For the experimental pulmonary metastasis model, 2×10^5^ B16 melanoma cells in 200μl PBS were injected into a lateral tail vein of each mouse. After 20 days, the mice were sacrificed and the metastatic foci of the lungs were quantified in each group under the dissecting microscope. Next, the lungs were paraffin-embedded, sectioned and stained with hematoxylin and eosin. Stained pulmonary sections were observed under the microscope.

### Platelet preparation, labeling and interaction with tumor cells

Mouse washed platelets were prepared as described [29]. Then, 4μM final concentration of calcein in DMSO was added to a suspension of 3×10^8^ platelets/ml in modified Tyrode buffer containing 5mM EDTA. Then the platelets were incubated for 30min at 37°C. Calcein-labeled platelets were collected by centrifugation, and the pellets re-suspended into modified Tyrode buffer.

To characterize tumor cell interaction with platelets, a total of 2×10^5^/ml, 6×10^5^/ml, or 1×10^6^/ml of Ds-Red transfected tumor cells in modified Tyrode buffer, respectively were added to 300μl suspensions of 3×10^8^ platelets/ml and stirred in Chrono-Log aggregometer at 1000rpm, 37°C. After 20 minutes, mixture of tumor cells and platelets was observed under bright field and fluorescence illumination.

To study the direct effects of platelets or recombinant TGF-β1 on adherent tumor cells, the tumor cells attached on plate were washed 3 times with preheated DMEM without serum and other supplements. Then, the washed cells were cultured for 48 hours in un-supplemented DMEM containing buffer, 20ng/ml of recombinant TGF-β1, or a final concentration of 2×10^8^/ml platelets. The morphology of tumor cells was observed under the phase-contrast microscope (Nikon).

### Cell invasiveness assay

To characterize of the role of the platelet ADP receptor P2Y12 in the invasiveness of LLC cells, the Matrigel-coated invasion assay was performed by adding 500μl suspensions of 2×10^4^ LLC cells with 2×10^8^/ml WT or P2Y12^−/−^ platelets in DMEM without serum to the upper surface of the transwell inserts, and 1ml DMEM without serum to the lower well of the assay chamber. Then, the plates containing the transwells were incubated at 37°C for 40 hours in a CO2 incubator. Next, the swabbed transwell inserts were placed into the wells containing 10% neutral formalin at room temperature for 30 min to fix the cells that invaded to the bottom surface of the porous membrane. The invasive cells were stained with crystal violet. After washing with PBS 3 times, the invasive cells were visualized, and counted using an inverted microscope (Nikon).

### Assay of the level of TGF-β1 by ELISA

After co-culturing tumor cells with platelets for 48 hours, the supernatant fractions were collected from the tumor cell conditioned medium, and the levels of active TGF-β1 were measured by using the immunoassay kit as directed by the manufacturer. Also, 300μl volumes of washed WT and P2Y12^−/−^ platelets, were stimulated by 20μM ADP, 2μg/mL collagen and 0.1U/ml thrombin, respectively. Then, 5mM EDTA was added and the supernatant fractions obtained by centrifugation. The level of active TGF-β1 released from the platelets was measured.

### Immunohistochemistry

Mouse lungs were fixed in 10% neutral formalin overnight. Then the fixed lungs were embedded with paraffin, and serial lung sections were prepared and stained with haematoxylin and eosin according to a standard protocol. For immunohistochemistry, sections were incubated in a humidified chamber with primary antibodies overnight at 4°C, the sections were treated with a rabbit monoclonal anti-mouse VEGFR1 IgG and a rabbit polyclonal anti-mouse fibronectin IgG to detect VEGFR1 and fibronectin positive areas in the lungs. Then, the sections were treated with a biotinylated anti-rabbit secondary antibody, followed with a streptavidin-peroxidase conjugate solution. Finally, the peroxidase substrate diaminobenzidine was added to display positive staining in the lungs. The number of VEGFR1 positive cells and cross-sectional areas of fibronectin positive staining regions were calculated using immunohistochemistry analysis software (KS400 version 3.0). A Zeiss-Axioplan2 microscope was used to visualize the images shown in the light micrographs.

### Statistical analysis

Data are presented as the mean ± SEM and analyzed using a 2-tailed Student’s t test for comparison of two independent groups with each other. Whereas, one-way ANOVA analysis were used for more than two independent groups using the GraphPad Prism Version 5.0 software.

## Results and Discussion

### Results

#### P2Y12 facilitates tumor metastasis in the LLC spontaneous pulmonary metastasis mouse model

**Figure 1:**
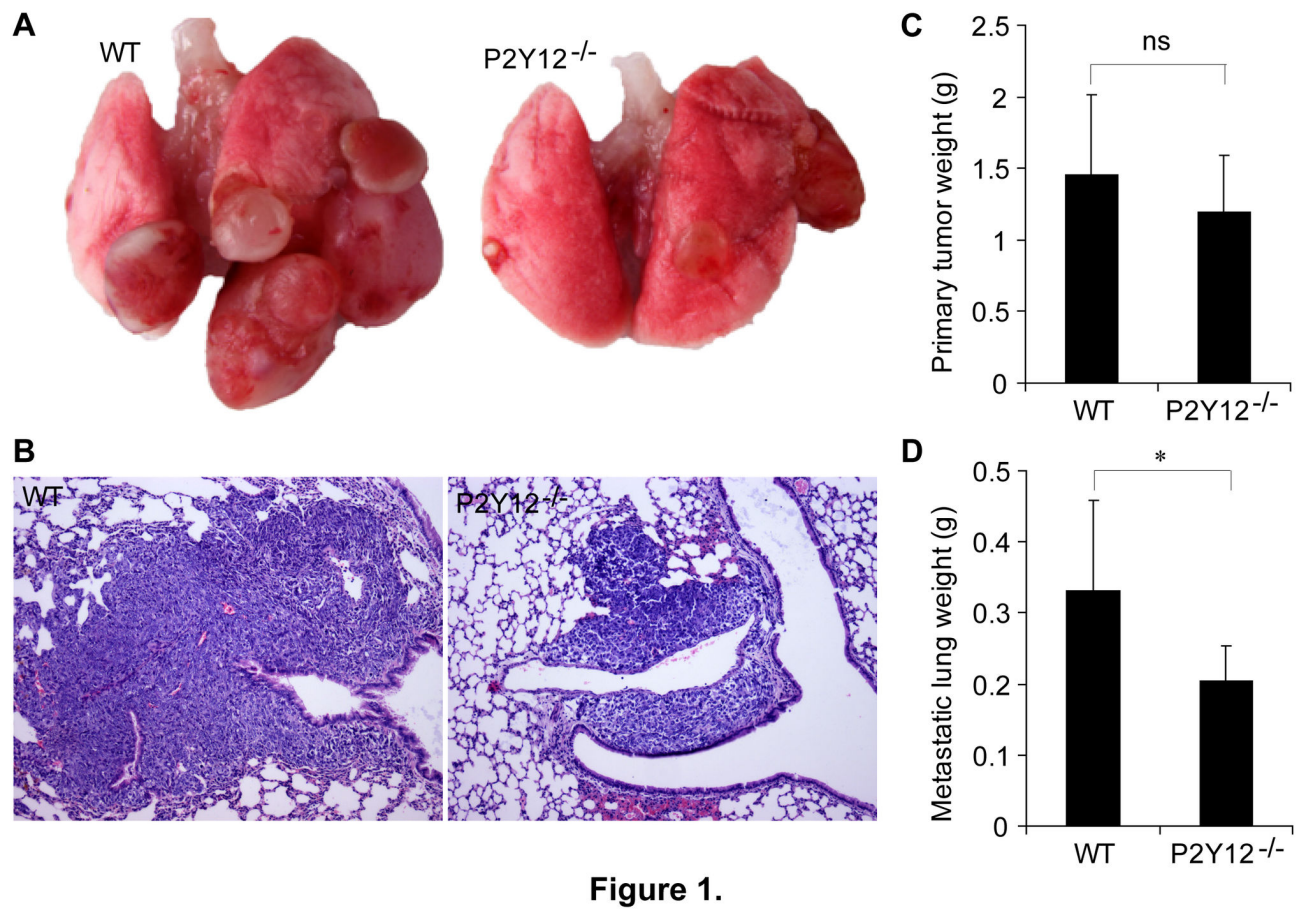
P2Y12 deficiency decreases lung metastasis but has no effect on primary tumor growth in the LLC (Lewis lung carcinoma) spontaneous pulmonary metastasis model. Each group of 9 mice was injected intradermally with 2×10^6^ LLC cells. Subcutaneous primary tumors were removed from each mouse and weighed 14 days after implantation. One month after removal of the subcutaneous tumors, each mouse was dissected, and its lungs were removed, weighed, and photographed. (**A**) Representative images of gross metastatic lungs from each group of mice. (**B**) Representative images of tissue sections from metastatic lungs obtained from WT and P2Y12 deficient mice. The lungs were paraffin-embedded prior to sectioning. The sections were stained with hematoxylin and eosin. (**C**) The mean weights of the primary tumors from the WT and P2Y12^−/−^ groups. Each bar represents the mean ± SEM of the weights of all the subcutaneous tumors from each group of mice. There was no statistically significant difference between the mean tumor weights of the WT and P2Y12 deficient groups (n=9). (**D**) The mean weights of metastatic lungs from WT and P2Y12^−/−^ groups. Each bar represents the mean ± SEM. P2Y12 deficiency significantly inhibited pulmonary metastasis (*p<0.05, n=9).

P2Y12^−/−^ and littermate control WT mice were used for the evaluation the effects of P2Y12 on tumor progression in the spontaneous pulmonary metastasis mouse model. Surgery was performed to remove the subcutaneous primary tumors at day 14 after intradermal implantation of the LLC cells. The weights of primary tumors were recorded. One month after surgery, the development of pulmonary metastatic foci was assessed in each group. Notably, the progression of metastasis in the lungs of the P2Y12^−/−^ mice was much less extensive than in the lungs of the WT mice (Figure 1A &1B). The mean weight of metastatic lungs was significantly less in P2Y12 deficient mice, than in the WT mice. The average value of metastatic lung weight in P2Y12^−/−^ mice was 0.21±0.02gram (g) compared with 0.33±0.06g in WT group (p<0.05; Figure 1D). This difference in the mean weight was the consequence of tumor burden difference, not smaller lungs with each group (Figures 1A & 1B). In contrast to metastasis, the growth of subcutaneous primary tumors was not affected by the absence of the P2Y12 receptor. The average weight of primary tumors in the P2Y12^−/−^ mice was 1.2±0.14g versus 1.45±0.19g in WT control mice (p>0.05; Figure 1C). These data suggest that P2Y12 is unable to increase primary tumor growth. In contrast, P2Y12 seems to be able to promote the spontaneous pulmonary metastasis.

#### The Ability of LLC Cells to Induce Platelet Shape Change Is Dependent on P2Y12

**Figure 2:**
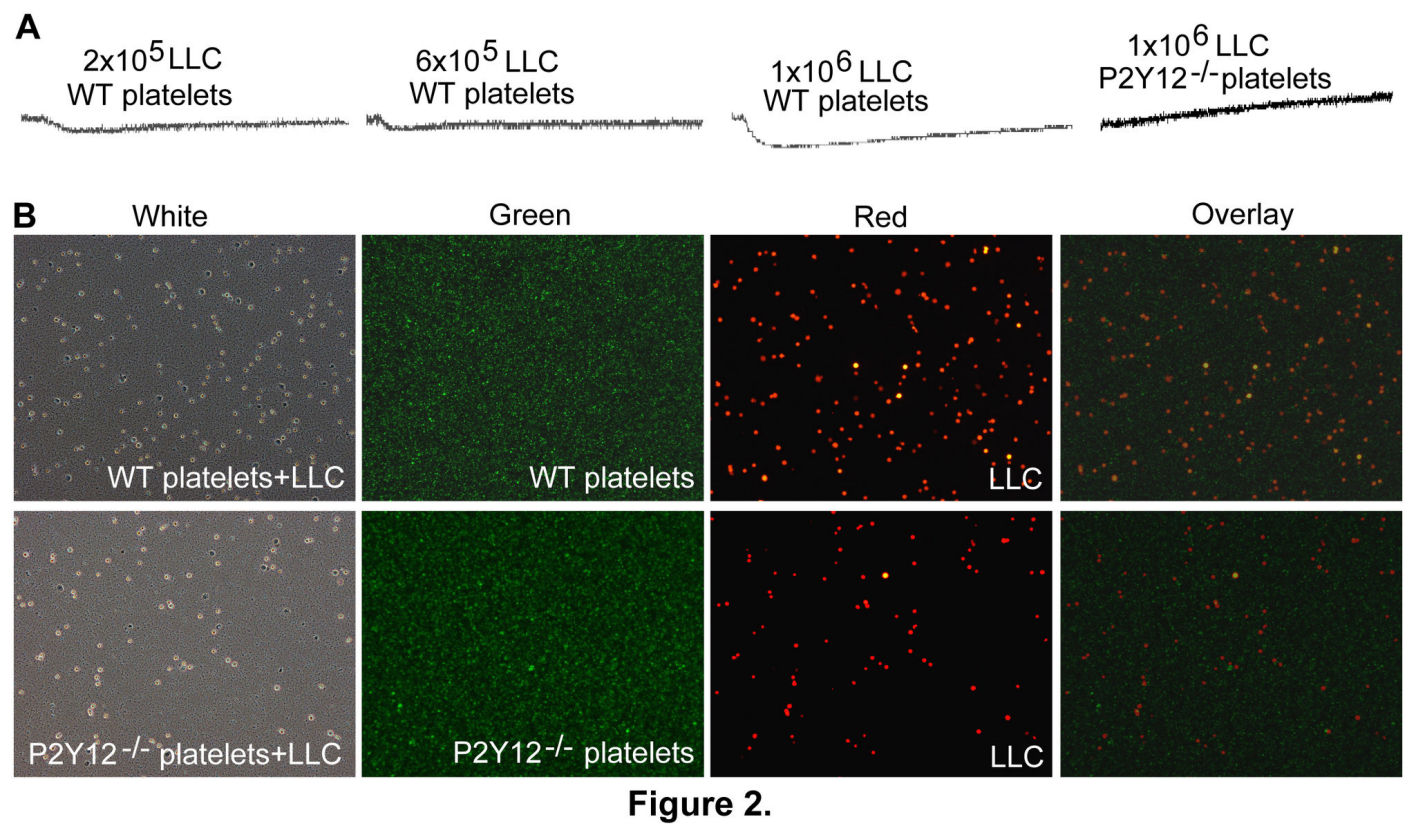
LLC cell induced platelet shape change is P2Y12-dependent. (**A**) In order to investigate the direct interaction between LLC tumor cells and platelets in vitro, 2×10^5^ /ml, 6×10^5^ /ml or 1×10^6^ /ml of Ds-Red labeled LLC cells were added to a suspension of 3×10^8^/ml calcein–labeled WT or P2Y12^−/−^ platelets in total volume of 300μl, and aggregation and shape change were monitored for 20 minutes with stirring at 1000rpm using a Chrono-Log aggregometeras described. In contrast to the WT platelets, P2Y12 deficient platelets did not undergo shape change or aggregate in response to stirring in the presence of LLC cancer cells (n=3). (**B**) After stirring, a sample of the mixture of LLC cells and platelets was smeared on the slides, and bright-field and fluorescence images of were taken in the 10x objective field. No obvious direct interaction between LLC cells and platelets was observed.

Several mechanisms for the involvement of platelets in tumor metastasis have been described, including tumor cell-platelet aggregation, as well as the metastasis-promoting effects of cytokines and chemokines secreted from activated platelets [30-33]. The interaction of LLC cells and platelets was studied. Interestingly, although LLC cells failed to induce platelet aggregation directly in vitro, they were able to induce platelet shape change. And, that effect was dependent on cell number and the P2Y12 receptor. The presence of 1×10^6^ tumor cells/ml in a 300μl suspension of 3×10^8^ platelets/ml could induce visible platelet shape change (Figure 2A). In an attempt to understand the mechanism of the ability of LLC cells to induce platelet shape change, the role of direct contact in this process was investigated. LLC cells stably expressing RFP fluorescent protein and calcein–labeled platelets were used for this purpose. Bright field and fluorescence micrographs failed to demonstrate the direct interaction between the LLC cells and platelets (Figure 2B). Therefore, the LLC cells apparently are able to induce platelet activation (shape change) through a non-contact, and P2Y12-dependent mechanism.

#### Platelets promote invasiveness of LLC cells by inducing a P2Y12 and TGF-β1 dependent epithelial-mesenchymal-like transition of those cells

**Figure 3:**
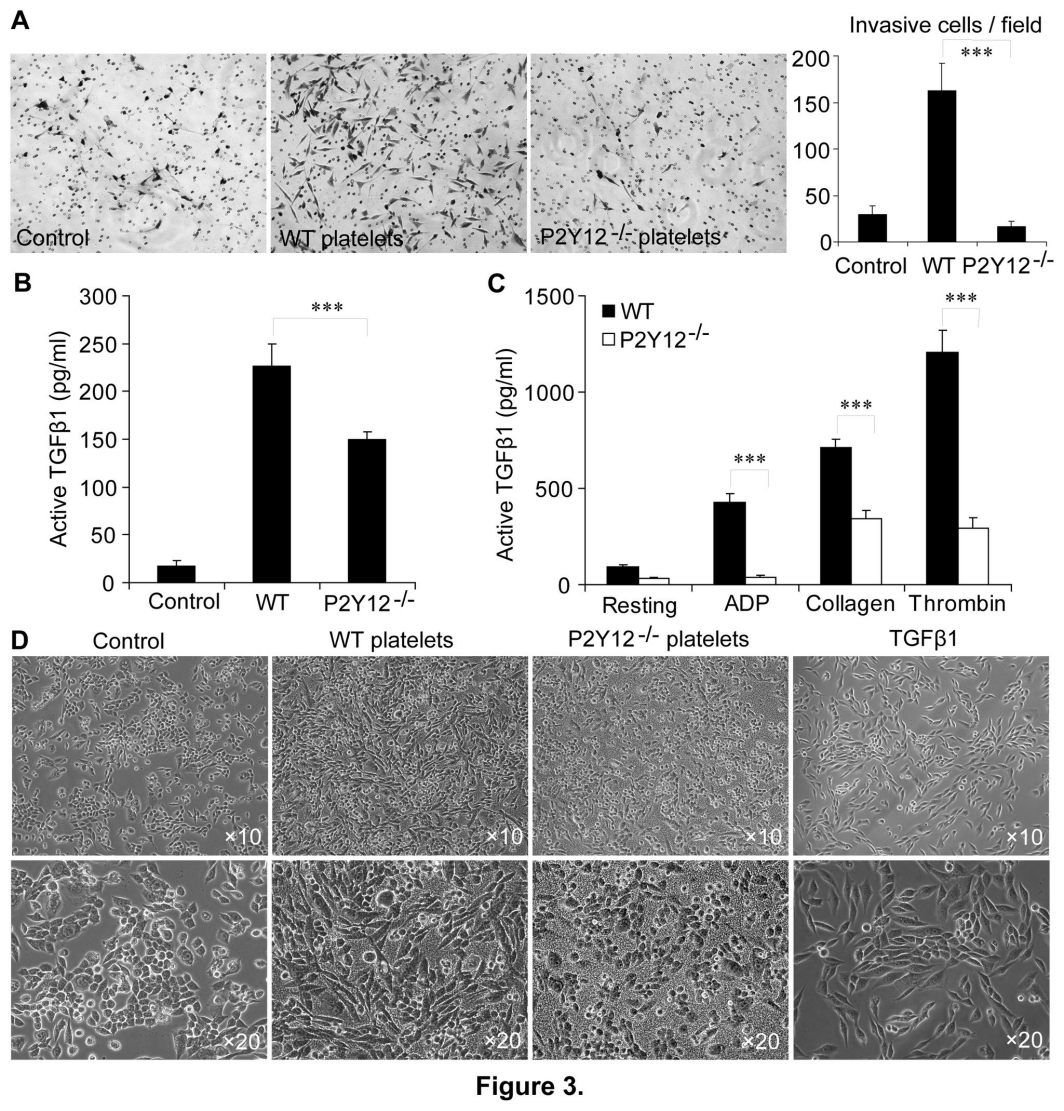
Platelets promote invasiveness of LLC cells by inducing a P2Y12 and TGF-β1 dependent epithelial-mesenchymal-like transition (EMLT). (**A**) Platelet P2Y12 regulated LLC cell invasion in matrigel. As evident from the images and bar graphs, P2Y12 deficient platelets significantly inhibited LLC cell invasiveness. LLC cells were added at the top of transwells coated with matrigel and those cells were treated with buffer, WT platelets and P2Y12^−/−^ platelets, respectively. The effects of this treatment were evaluated by counting the crystal violet stained cells at the bottom of the wells after 40 hours incubation with buffer or platelets. Each bar represents the mean number of invasive cells ± SEM, statistical significance was determined using a one-way AVONA (10 random fields per sample, n=3 samples, ***p<0.001). (**B**) LLC cells induced the release of active TGF-β1 from platelets. The LLC cells attached to each well were incubated with buffer, WT or P2Y12^−/−^ platelets for 48 hours. Cancer cells and platelets were removed from the conditioned medium by centrifugation. The amounts of the active form of TGF-β1 in the supernatant fractions were measured using ELISAs. Each bar represents the mean ± SEM, and the statistical significance was calculated using a one-way AVONA (***p<0.001, n=3). (**C**) The levels of active TGF-β1 were measured in the supernatant fractions from 300μl volumes of washed platelets stimulated by 20μM ADP, 2μg/ml collagen or 0.1U/ml α-thrombin. EDTA (final concentration of 5mM) was added to each platelet suspension before the supernatant fractions were prepared by centrifugation. The level of TGF-β1 was measured in each supernatant fraction using an ELISA. Each bar represents the mean ± SEM, and the statistical significance was calculated using a one-way AVONA (***p<0.001, n=3). (**D**) 10× and 20× phase-contrast micrographs of LLC cells that had been incubated with buffer, WT platelets, P2Y12^−/−^ platelets or 20ng/ml recombinant active TGF-β1 (as a positive control). In contrast to the P2Y12 deficient platelets, incubation of the LLC cells with WT platelets or active TGF-β1 obviously induced the LLC cells to undergo an EMT-like transition.

The ability of platelets to enhance LLC tumor cell invasiveness was investigated as described above using the Matrigel-coated transwell system [34]. For this purpose, 2×10^4^ LLC cells were incubated for 40 hours at 37°C in the upper surface of each Matrigel-coated transwell in the presence of WT or P2Y12^−/−^ platelets. Platelets from the WT mice, but not from the P2Y12^−/−^ mice significantly increased the invasiveness of the LLC cells (Figure 3A). Specifically, the mean count of invasive LLC cells was significantly reduced from 163±10/field in the wells containing the WT platelets to 17±2/field in the wells containing the P2Y12^−/−^ platelets (P<0.001; Figure 3A). These results indicate that platelet P2Y12 regulates tumor cells invasiveness.

Recent studies have resulted in the idea that platelets are able to induce tumor cells to undergo an epithelial-mesenchymal-like transition and thereby promote tumor cell invasiveness [11]. Induction of an EMT-like transition presumably involves TGF-β1 signaling [14]. Because circulatory TGF-β1 is mainly derived from the platelet granules [11], we investigated whether or not P2Y12 affects tumor cell metastasis by enhancing TGF-β1 secretion from activated platelets. Equivalent volumes of Tyrode buffer, a suspension of 2×10^8^/ml of WT and P2Y12^−/−^ platelets, respectively were added to cultured LLC cells. After incubation of the platelet/cell mixtures for 48 hours at 37°C, the levels of active TGF-β1 in the conditioned medium were 17.6±3.4, 227.2±13.2 and 150.3±4.3 pg/ml for the treatments with buffer, WT or P2Y12^−/−^ platelets (P<0.001; Figure 3B). These results confirmed previous reports [11] and demonstrated that P2Y12 enhances the release of active TGF-β1 from platelets in response to incubation with tumor cells. This conclusion was consistent with the effect of P2Y12 deficiency on the secretion of active TGF-β1 from platelets stimulated by the agonists ADP, collagen and thrombin, respectively (P<0.001; Figure 3C). Moreover, LLC cells underwent a spindle-like, fibroblastic morphological change, reminiscent of an EMT-like transition in response to incubation with the WT platelets for 48 hours, just as the LLC cells did in response to treatment with 20ng/ml of recombinant, active TGF-β1. As expected, LLC cells incubated with either P2Y12-deficient platelets or Tyrode solution did not undergo an EMT-like morphological change (Figure 3D). TGF-β1 neutralized antibody significantly blocked platelets induced EMT-like morphology change of LLC, suggesting TGF-β1 is the major cytokine from platelets to cause LLC EMT-like morphology change (Figure S1). All of these results support the conclusion that the ADP receptor P2Y12 facilitates platelet secretion of active TGF-β1 which in-turn increases tumor cells invasiveness by apparently promoting an EMT-like morphological change of the tumor cells.

#### P2Y12 is involved in pre-metastatic niche formation

**Figure 4:**
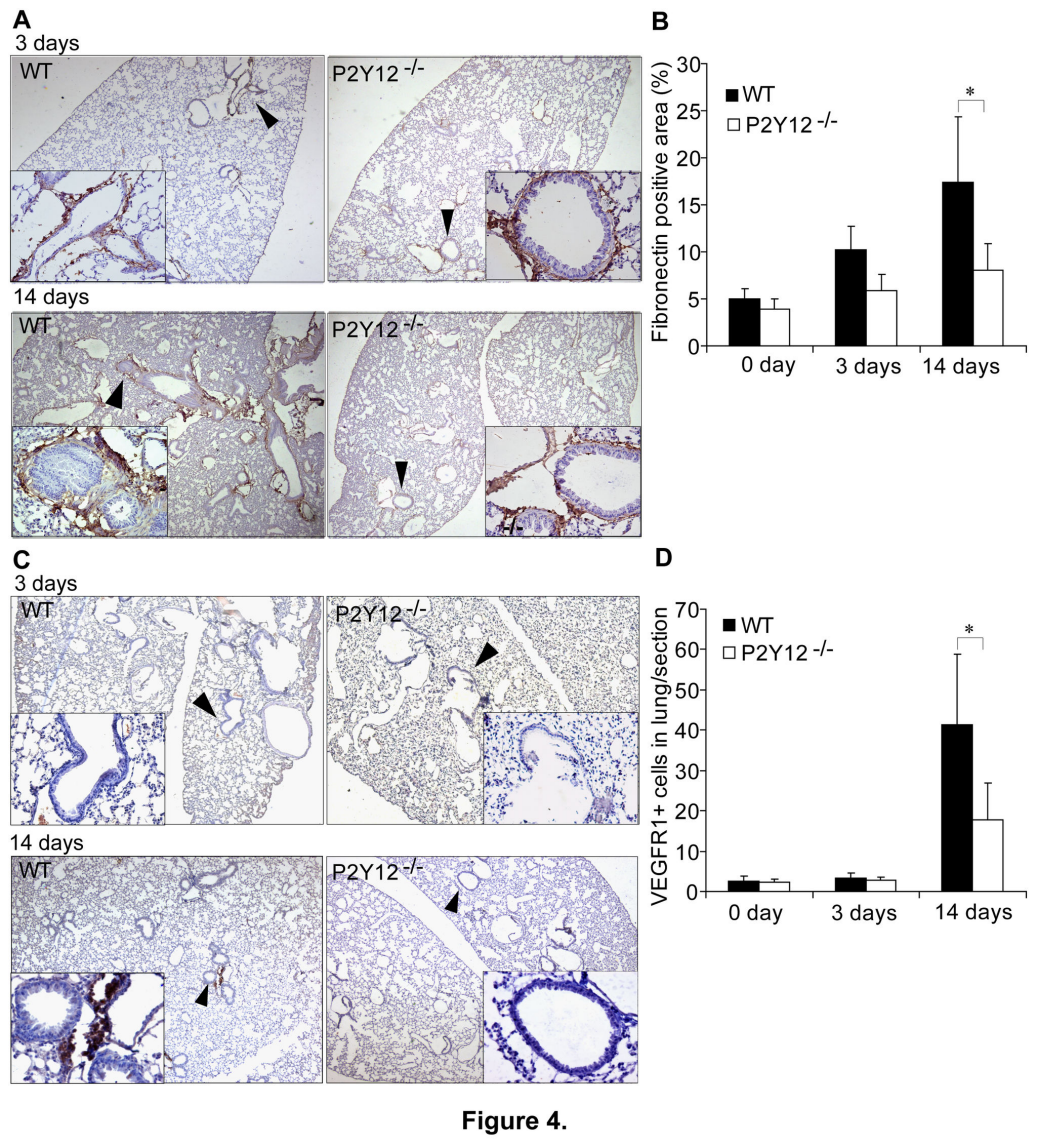
P2Y12 regulates/affects the formation of the pre-metastatic microenvironment in the lungs. (**A**) Representative images of fibronectin expression in lung sections. The levels of tissue fibronectin were measured in lung sections prepared at 3 and 14 days after intradermal implantation of the LLC cells. Immunostaining analysis was used to measure tissue fibronectin. The levels of lung tissue fibronectin were obviously enhanced at day 14 in the WT group, but not in the P2Y12 deficient group. In the latter group, fibronectin was expressed mainly in the regions of micro-vessels and terminal bronchi. Arrows indicate the fibronectin positive areas in the lungs of each group, and enlarged insets in 40× magnification are shown. (**B**) Statistical analyses of the percentages of fibronectin positive areas in sections of the total lungs for all the animals in each group. The lungs from both groups of animals were approximately the same size. P2Y12 deficiency did not support metastasis-driven enhancement of fibronectin expression (3 sections for each mouse; n=5 for each group, *p<0.05). (**C**) The numbers of VEGFR1 positive cells were measured in lung sections prepared on days 3 and 14 after intradermal injection of the LLC cells. By day 14, VEGFR1^**+**^ cell clusters were apparent surrounding the distal bronchials and microvascular regions of the WT mouse lungs. In contrast, the P2Y12 deficient mice had few VEGFR1+ cell clusters in their lungs. Arrows indicate the VEGFR1^**+**^ cell clusters in the lungs of each group, and enlarged 40× insets in are shown. (**D**) Statistical analyses of the mean number of VEGFR1^**+**^ cells per lung section P2Y12 deficiency did not support metastasis-driven recruitment of VEGFR1^**+**^ cells to the lungs. Each bar represents the mean ± SEM, and statistical significance was determined using a one-way AVONA (3 sections for each mouse; n=5 for each group, *p<0.05).

Fibronectin, the major component of extracellular matrix and an integrin-ligand that is involved in extracellular remodeling, wound healing and chronic inflammation, and supports bone marrow-derived cell (BMDCs) mobilization and cellular adhesion [35,36]. Also, fibronectin is up-regulated strongly in the connective tissue of a pre-metastatic organ, providing a permissive niche for the arrival of metastatic tumor cells [35]. Immunohistochemical analyses of lung samples from LLC cell-treated mice showed an increased fibronectin expression from day 3 following intradermal implantation of the LLC cells (Figure 4A) to a maximal deposition by day 14 after implantation (Figure 4A). The increased deposition occurred in the peribronchial and microvessel regions in the WT mice, but did not occur in the P2Y12^−/−^ mice (p<0.05; Figure 4A and B).

The importance of the involvement of bone marrow-derived cells (BMDCs) in tumor development has been well documented recently [37-39]. Vascular endothelial growth factor receptor 1 positive (VEGFR1+) BMDC clusters are recruited to the pre-metastatic sites before the arrival of LLC cells in the spontaneous metastasis model [35]. It has been reported that cytokines and chemokines released from activated platelets can mobilize BMDCs to repair injured tissues [40] and affect tumor progression [41]. Therefore, we speculated that P2Y12 might affect tumor metastasis by facilitating the establishment of VEGFR1+ BMDC clusters in the pre-metastatic microenvironment. Consequently, the recruitment of VEGFR1 positive cells into the lungs of WT and P2Y12^−/−^ mice, following intradermal injection of LLC cells was investigated to test this idea. Immunohistochemical analyses of lung tissues harvested at day 14 after tumor implantation revealed an obvious recruitment of VEGFR1 positive cell clusters near the terminal bronchioles and distal alveoli, before the arrival of tumor cells in WT mice (Figure 4C). But, such recruitment was attenuated in P2Y12^−/−^ mice (p<0.05; Figure 4C and D), despite no marked differences between the lungs at day 3 (Figure 4C and D).

Therefore, P2Y12 appears to enhance pre-metastatic niche formation; whereas, P2Y12 deficiency does not support enhanced ECM fibronectin deposition and the recruitment of VEGFR1 positive bone marrow-derived cells to targeted lungs, and therefore does not support metastatic niche formation.

#### B16 cells induce platelet aggregation in a cell number and P2Y12-dependent manner

**Figure 5:**
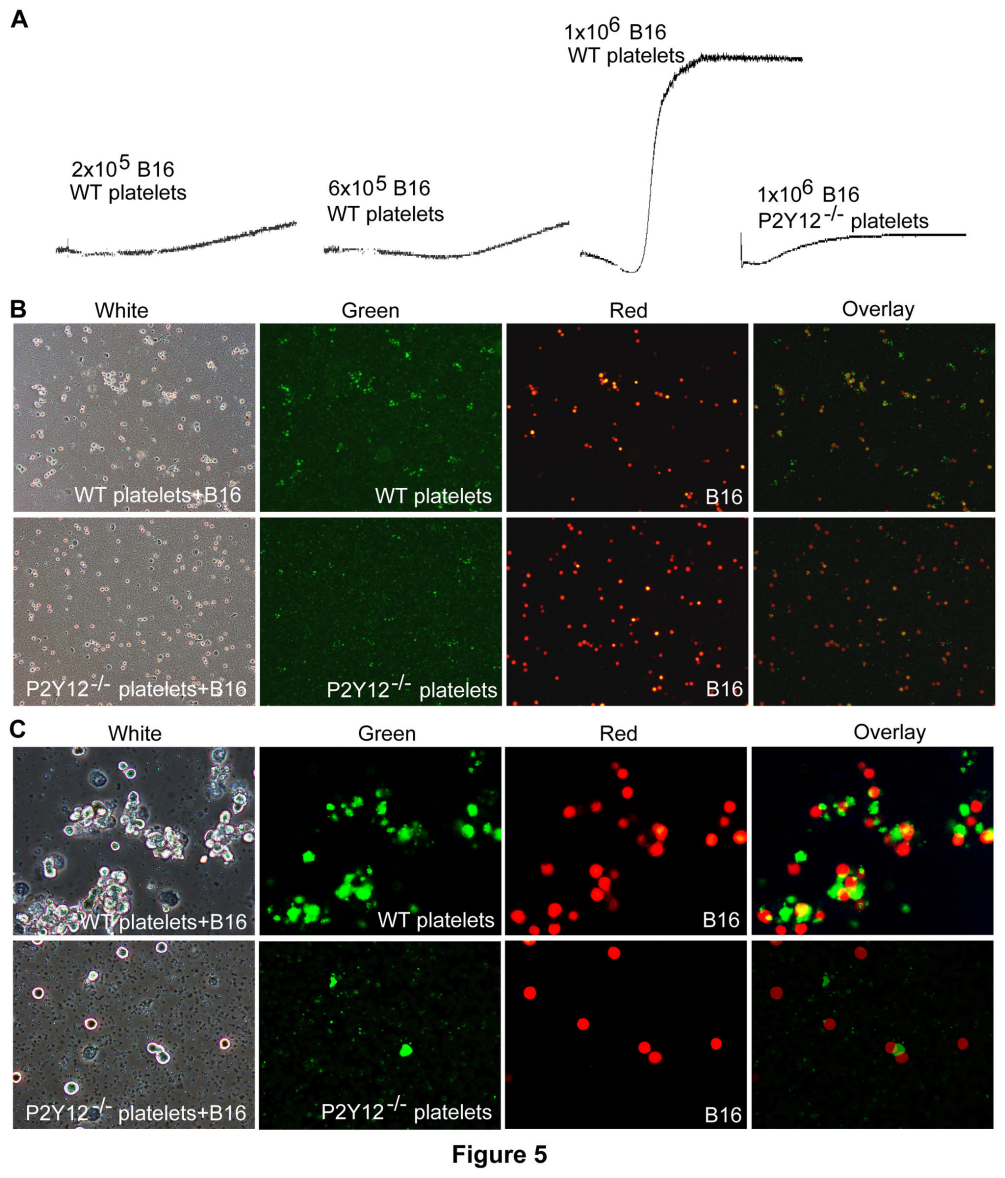
B16 cells induced platelet aggregation in a cell number and P2Y12-dependent manner. (**A**) In order to examine the ability of B16 melanoma cells to directly cause platelet aggregation in vitro, 300μl suspensions containing 2×10^5^/ml, 6×10^5^/ml or 1×10^6^/ml of Ds-Red labeled B16 cells, respectively and 3×10^8^/ml calcein–labeled WT or P2Y12^−/−^ platelets were prepared, and aggregation was monitored using a Chrono-Log aggregometer. B16 melanoma cells were able to induce visible platelets-cancer cells aggregates. The aggregation was dependent on cancer cell number and WT platelets, and consequently was not supported by P2Y12 deficient platelets (n=3). (**B** & **C**) In order to detect the direct interaction between the B16 melanoma cells and the platelets in vitro, after the 20 minute aggregation trial, aliquots of each mixture of B16 cells and platelets were smeared on glass slides, and bright-field and fluorescent images were recorded at 10× and 20× magnifications. The images clearly revealed that some of the B16 cells directly interacted with WT platelets resulting in platelet accumulation on the surface of the B16 cells. In contrast, the P2Y12 deficient platelets did not support extensive accumulation of platelets on the surface of B16 cells (n=3).

Although our data demonstrate that P2Y12 plays an important role in pulmonary epithelial-originated LLC cell metastasis, it is unknown whether or not P2Y12 is also able to regulate the metastasis of non-epithelial tumor cells. Accordingly, the role of P2Y12 in the metastasis of murine-derived melanoma B16 cells was investigated. Specifically, 300μl suspensions of 1×10^6^ B16 tumor cells/ml mixtures containing 3×10^8^ platelets/ml stirred in a Chrono-Log aggregometer at 1000 rpm, for 20 minutes at 37°C, caused a maximum of fifty percent aggregation. In contrast, under similar conditions, mixed suspensions of P2Y12-deficient platelets with B16 cells did not induce platelet aggregation (Figure 5A). The basis of the B16-induced aggregation of WT platelets was investigated using fluorescence microscopy. Moreover, 1×10^6^/ml of Ds-Red B16 cells stably expressing a red fluorescent protein were added to suspensions of calcein–labeled WT and P2Y12^−/−^ platelets, respectively and aggregation was examined in the aggregometer. Microscopy images revealed that the B16 cells formed mixed aggregates of tumor cells and platelets in the presence of WT platelets, but not in the presence of P2Y12^−/−^ platelets (Figure 5B). Although the P2Y12-deficient platelets apparently did not support B16-induced mixed aggregation of B16 cells and platelets in the aggregometer, sporadic direct interaction between platelets and rare B16 cells was apparent using microscopy (Figure 5C). Therefore, B16 melanoma cells could directly interact with WT platelets and cause them to aggregate in a manner that is dependent on both P2Y12 and the number of B16 cells.

#### P2Y12 facilitates B16 cells to undergo a TGF-β1-independent EMT-like transition, and experimental pulmonary metastasis

**Figure 6:**
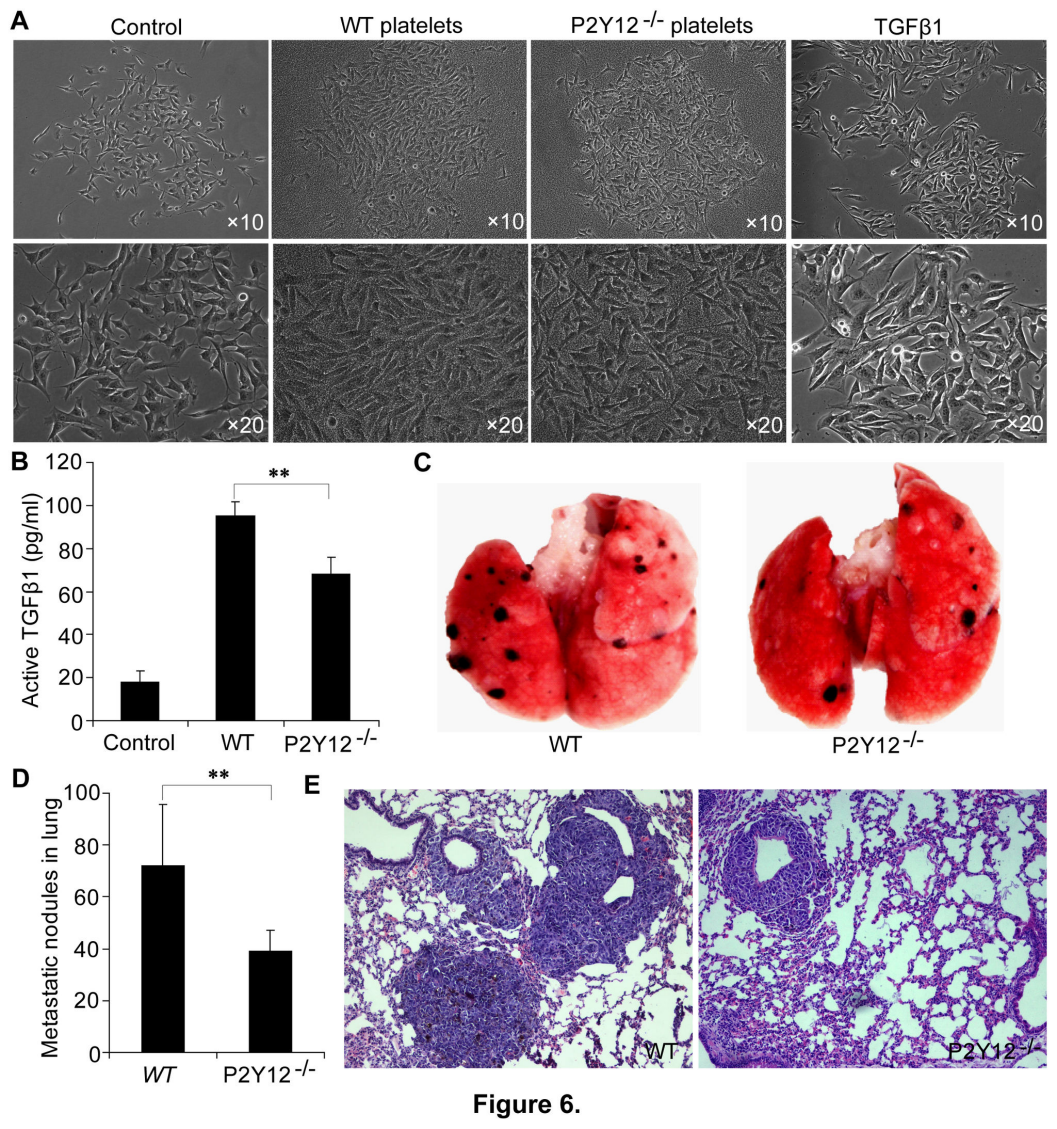
P2Y12 facilitates a TGF-β1-independent EMT-like transition of B16 cells, and experimental pulmonary metastasis by B16 cells. (**A**) Phase-contrast 10× and 20× micrographs of B16 tumor cells incubated with buffer, WT platelets, P2Y12^−/−^ platelets or 20ng/ml active TGF-β1, respectively for 48 hours. Incubation with WT platelets obviously induced B16 cells to undergo an EMT-like transition, P2Y12 deficient platelets did not induce an EMT-like transition of B16 cells. Interestingly, the recombinant active TGF-β1 induced the B16 cells to undergo a less extensive EMT-like morphologic change than was induced by the WT platelets. (**B**) The level of active TGF-β1 in the B16 conditioned medium was also measured by an ELISA. B16 cells induced significantly less release of active TGF-β1 from P2Y12 deficient platelets than from WT platelets. Each bar represents the mean ± SEM, and statistical significance was determined using a one-way AVONA (**p<0.01, n=3). (**C**) Each mouse from both groups (n=7 for each group) were injected with 2 × 10^5^ B16 cells via a tail vein. Twenty days after injection of the B16 cells, the, lungs were dissected from each mouse, and photographed. Images of visible metastatic foci are apparent in the photomicrographs. (**D**) Statistical analyses of the number of metastatic foci at the surface of the lung lobes. Each bar represents the mean ± SEM, and statistical significance was determined using the Student’s t test (**p<0.01, n=7 for each group). (**E**) Representative histochemical images (10×) of lung sections from wild-type and P2Y12 deficient mice.

Consistent with results obtained from the LLC cells, B16 cells underwent an EMT-like transition upon incubation with WT platelets, but not with P2Y12-deficient platelets (Figure 6A). Similarly, ELISA based measurements revealed increased levels of active TGF-β1 in the conditioned medium from co-cultures of B16 cells and WT platelets compared with medium from cells cultured in the absence of platelets. Also, the concentration of active TGF-β1 was markedly diminished in co-cultures of cells with P2Y12^−/−^ platelets (p<0.01; Figure 6B). Interestingly, the EMT-like morphologic change of B16 cells induced by recombinant active TGF-β1 was substantially less pronounced than that induced by incubation with WT platelets for 48 hours. Those results suggested that the P2Y12-dependent regulation of the EMT-like morphologic change of the melanoma B16 cells is not TGF-β1 specific.

We next investigated whether or not P2Y12 affects B16 tumor cell metastasis in vivo. For this purpose, 2×10^5^ B16 cells were injected into each mouse of both groups via a tail vein. Quantitative evaluation the number of pulmonary metastatic loci was performed at day 20 after injection. The results showed that P2Y12^−/−^ mice had significantly fewer metastatic foci in their lungs than developed in the lungs of the WT mice (Figure 6C & 6E). The average number of metastatic foci in the P2Y12 deficient mice was 39±3 foci/lung. In contrast, the WT mice had an average number of 72±8 foci/ lung (two lobes, P<0.01; Figure 6D). Together, these results demonstrate in contrast to P2Y12, P2Y12 deficiency does not support experimental pulmonary metastasis by B16 cells.

### Discussion

P2Y12 plays a crucial role in platelet alpha-granule secretion, P-selectin expression, and integrin αIIbβ3 activation [19]. Though all of these characters correlate with metastasis [19], the relationship between P2Y12 and tumor progression had not been elucidated.

In this study, we found that murine pulmonary epithelial-originated LLC cells were able to induce platelet shape change by a cell number and P2Y12-dependent mechanism that is not dependent on direct tumor cell-platelet interaction (Figure 2A). On the contrary, murine non-epithelial melanoma B16 cells could directly interact with platelets and cause them to aggregate in a manner that is dependent on both P2Y12 and the number of B16 cells. Therefore, the mechanism of tumor cell induced platelet activation is dependent on the type of tumor cell, cell number and platelet P2Y12. But elucidation of the molecular bases of LLC cell and B16 cell induced platelet activation requires further investigation, despite the published results of melanoma cell-induced platelet aggregation [42].

Epithelial-mesenchymal transitions (EMT) occur as key steps during embryonic morphogenesis, and are now implicated in tumors progression [43]. A recent study has proved that TGF-β1 released from activated platelets contributes to EMT-like morphological changes of tumor cells and thereby promotes tumor cell invasiveness [11]. The results presented here in Figure 3 confirmed previous reports that tumor cells can induce platelet release of TGF-β1, and further demonstrated that P2Y12 enhances TGF-β1 release from platelets in response to exposure to tumor cells. This conclusion was consistent with the effect of P2Y12 deficiency on the secretion of active TGF-β1 from platelets stimulated by agonists ADP, collagen and thrombin, respectively. Morphological observations indicated that LLC cells co-cultured with WT platelets or recombinant TGF-β1, but not P2Y12-deficient platelets, underwent an EMT-like change. Matrigel-coated transwell experiments further demonstrated that platelet P2Y12 promotes the invasiveness of LLC cells. Therefore, the ADP receptor P2Y12 facilitates platelet secretion of active TGF-β1, that in-turn increases LLC cell invasiveness by apparently promoting an EMT-like change of the tumor cells. As with the LLC cells, B16 melanoma cells also underwent an EMT-like change in presence of WT but not P2Y12-deficient platelets. However, recombinant TGF-β1 induced B16 cells to undergo a very modest spindle-like morphological change, a morphological change less apparent than that resulting from incubation of the cells with WT platelets. Therefore, the P2Y12-dependent regulation of the EMT-like morphologic change of the melanoma B16 cells presumably is not TGF-β1 specific. This is the case even though P2Y12 promotes B16 cell induced TGF-β1 release from platelets. So, further work is required to elucidate the molecular mechanisms that underlie the ability of platelet P2Y12 to promote B16 melanoma cells to undergo an EMT-like change.

In rodent models, an appropriate pre-metastatic tumor microenvironment enhances metastasis [35]. Fibronectin, a major component of the extracellular matrix and an integrin-ligand, is up-regulated strongly in the connective tissue of pre-metastatic organs, providing a permissive niche for the arrival of metastatic tumor cells [35]. In this study, immunohistochemical analyses of lung samples showed increased lung tissue fibronectin in the WT mice following the implantation of LLC cells, but a similar fibronectin increase did not occur in the LLC cell treated P2Y12^−/−^ mice. As with fibronectin, bone marrow-derived cells (BMDCs) play a role in tumor development [37-39]. VEGFR1 positive BMDC clusters are recruited to the fibronectin enriched lung tissue sites and form pre-metastatic niches to facilitate metastasis of LLC cells in the spontaneous pulmonary metastasis model. Specifically, immunohistochemical analyses of lung tissues harvested during day 14 after tumor implantation revealed an obvious recruitment of VEGFR1 positive cell clusters before the arrival of tumor cells in WT mice. But, such recruitment was attenuated in P2Y12^−/−^ mice, despite no marked differences between the lungs of the two strains at day 3. Therefore, P2Y12 appears to enhance pre-metastatic niche formation and therefore metastasis; this is apparent because in the absence of P2Y12 function, little enhancement of lung tissue fibronectin or the recruitment of VEGFR1 positive bone marrow-derived cell clusters to the targeted lungs occurred. In other words, the absence of P2Y12 function did not support pre-metastatic niche formation. Furthermore, cytokines and chemokines released from platelet α-granules can mobilize BMDCs to repair injured tissues [40,41], and are also involved in angiogenesis [40,41]. Since α-granule release is P2Y12 dependent, platelet P2Y12 probably mediated the recruitment of VEGFR1 positive BMDCs to the pre-metastatic niche through regulating the release of cytokines from α-granules. The detailed mechanisms need further investigation.

Our results clearly show that P2Y12 deficiency suppress the metastasis in both mouse models. Assuming similar effects of the absence of P2Y12 function in humans and no serious off target effects of P2Y12 antagonists, our data provide justification for considering the use of P2Y12 antagonists as anti-metastasis agents in pre-metastasis cancer patients with a platelet-based thrombotic disorder.

## Supporting Information

Figure S1 **LLC cells were incubated with WT platelets in absence or presence of TGF-β1 neutralized antibody (6μg/ml) for 48 hours at 37°C.** TGF-β1 neutralized antibody significantly blocked platelets induced EMT-like morphological change of LLC cells.
(DOCX)
