# Supplementary material for: Platelet P2Y12 Is Involved in Murine Pulmonary Metastasis
Source: PLoS One. 2013 Nov 13;8(11):e80780. doi: 10.1371/journal.pone.0080780 (PMC3827483; doi:10.1371/journal.pone.0080780)
Supplement: Figure S1 — LLC cells were incubated with WT platelets in absence or presence of TGF-β1 neutralized antibody (6μg/ml) for 48 hours at 37°C. TGF-β1 neutralized antibody significantly blocked platelets induced EMT-like morphological change of LLC cells. (DOCX) [file pone.0080780.s001.docx]

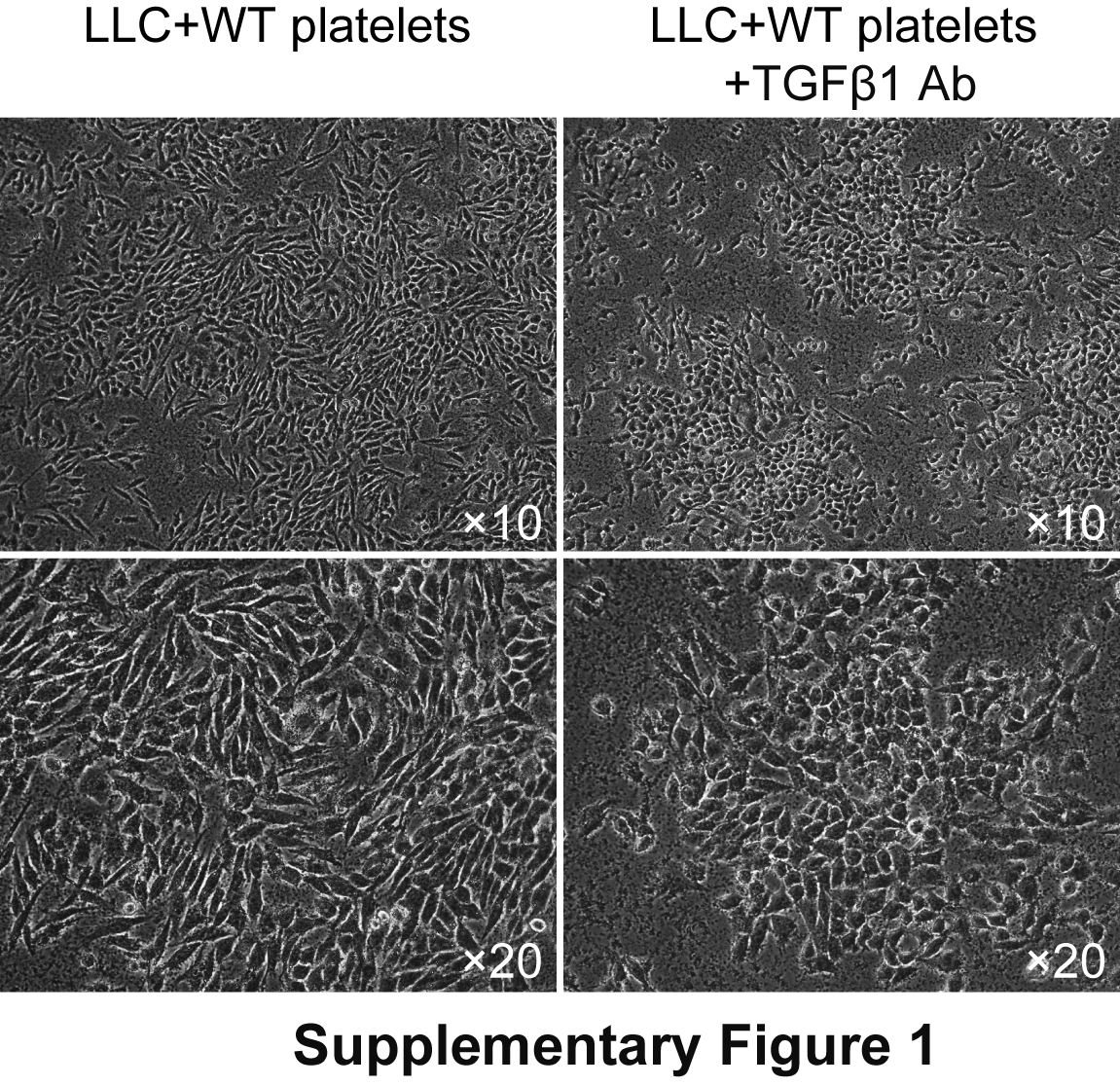


**Supplementary Figure 1:** LLC cells were incubated with WT platelets in absence or presence of TGFβ1 neutralized antibody (6ug/ml) for 48 hours at 37℃. TGFβ1 neutralized antibody significantly blocked platelets induced EMT-like morphology change of LLC.
